# Enhancing the Emulsification and Photostability Properties of Pectin from Different Sources Using Genipin Crosslinking Technique

**DOI:** 10.3390/foods11162392

**Published:** 2022-08-09

**Authors:** Jiawei Lin, Hecheng Meng, Xiaobing Guo, Shujuan Yu

**Affiliations:** 1 School of Food Sciences and Engineering, South China University of Technology, Guangzhou 510640, China; 2 School of Food Science and Technology, Shihezi University, Shihezi 832003, China

**Keywords:** sugar beet pectin, citrus pectin, apple pectin, genipin, emulsification performance, photostability

## Abstract

Pectin is a potential polysaccharide-based emulsifier, but the stabilized emulsions suffer from insufficient emulsion stability. Therefore, modification is needed to enhance its emulsification performance to cater to practical applications. The genipin-crosslinking strategy was used in this work to modify pectin with different sources and extraction conditions. Chemical composition analysis, molecular weight (Mw), and radius of gyration (Rg) measurement revealed that sugar beet pectin (SBP) has a more compact and flexible conformation than commercial citrus pectin (CP) and apple pectin (AP), indicated by the significantly (*p* < 0.05) larger Mw/Rg of SBP (18.1–11.3 kg/mol/nm) than CP (8.3 kg/mol/nm) and AP (8.0 kg/mol/nm). Crosslinking modification significantly increased the Mw, radius of gyration, and viscosity. This significantly (*p* < 0.05) improved the emulsifying stability (a smaller increase in droplets size) by the contribution of both thicker adsorbed hydrated layers at the oil-water interface with a stronger steric-hindrance effect and larger viscosity effect to slow down droplet collision. The formation of a blue-black substance from crosslinking reaction was able to block the UV radiation, which significantly improved the photostability of *β*-carotene-loaded emulsions. Altogether, genipin-crosslinking is feasible to modify pectin of different sources to enhance the emulsion stability and for use as a vehicle for delivering bioactive compounds.

## 1. Introduction

Pectin mainly exists in the primary cell wall and the intercellular layer of the plant. It is a family of heteropolysaccharides backboned with homogalacturonan (HG) and inserted by rhamnose as the branch to link with galactan and arabinogalactan as side chains. A small amount of protein and phenol (i.e., ferulic acid) is bound to the carbohydrate by ester linkage [1]. The common use of pectin in the food industry is as a gelling agent, stabilizer, and viscosity enhancer. Pectin gel is formed with a high concentration of sugar at acid pH (<3–4) or added with calcium ions.

Recently, the emulsifying ability of pectin attracted great attention due to the label-clean demand for naturally occurring emulsifiers. Extensive studies have manifested the emulsification mechanism of pectin: the covalently bound hydrophobic proteinaceous moiety adsorbed on the oil-water interface as an anchor, whilst the hydrophilic polysaccharide chains extended into the continuous phase as a bulky hydrated layer to prevent the emulsion from coalescence [2]. Pectin originated from different sources exhibited inconsistent emulsification performance. Although sugar beet pectin (SBP) does not form a gel, the high protein content and side chains endow it with superior emulsifying properties to citrus pectin (CP) and apple pectin (AP) [3]. Compared to gum Arabic (the “gold standard” emulsifier in the food industry) [4,5], SBP has the advantage of stabilizing emulsion at a relatively lower dosage, which is a promising polysaccharide-based emulsifier [6]. However, the undesirable emulsifying stability (especially after thermal sterilization) greatly restricted the application of SBP [7]. In addition, the variation of batches and extraction methods will lead to great variability in the emulsification of SBP.

By polymerizing modification (enzymatic crosslinking [7], thermal “maturation” modification [8]), it was found that the increase of molecular weight (Mw) would significantly thicken the hydrated layer at the oil-water interface, therefore enhancing the emulsifying stability by stronger steric-hindrance effect. In our previous work, a genipin-crosslinking strategy could substantially enlarge the Mw of SBP and fabricate emulsion with long-term stability [9]. However, the initial pectin used for polymerization in the existing studies is excellent in emulsifying activity for improving emulsifying stability. Whether pectin of different batches and different sources with varying emulsifying activities can be polymerized to enhance emulsification is still unknown.

One of the potential applications of emulsion is used to deliver the active component [10]. Many active components are photosensitive, especially to ultraviolet (UV) light [11,12]. Adding antioxidant substances into the emulsion and adopting an opaque container can improve the photostability of the photosensitive substances in the emulsion [13]. Studies have shown that the blue substance produced after the crosslinking of ginipin can absorb part of the UV light, thus improving the UV resistance of montmorillonite-chitosan film [14]. Our previous study also found that the color of pectin changed from light yellow to dark blue after being crosslinked with genipin, making the fabricated emulsions appear blue color [9,15]. We speculate that the blue substance and the thicker hydrated layer at the oil-water interface resulting from genipin-crosslinking modification can facilitate the photostability of emulsion.

In the present study, we first characterized and crosslinked different SBP samples and pectin of different sources, including the high methoxy pectin (extracted SBP1 and commercial CP and AP) and low methoxy SBP2 and SBP3. Next, the modification was verified by Mw measurement and viscosity test. Finally, the emulsification performance and photostability to protect *β*-carotene were examined. This work provided an in-depth understanding of using the genipin-crosslinking strategy to enhance the emulsification of pectin of different sources and extraction conditions.

## 2. Materials and Methods

### 2.1. Materials

Sugar beet pectin was donated by Lvxiang Beet Sugar Co., Ltd. (Urumchi, China). Medium-chain triglyceride (MCT) was purchased from Britz Networks Sdn. Bhd (Melaka, Malaysia). *β*-carotene (purity ≥ 95%), ferulic acid (FA, purity ≥ 99%), CP (galacturonic acid ≥ 65%, degree of methylation ≥ 50%), and AP (galacturonic acid ≥ 72%, degree of methylation ≥ 65%) were the products of Sigma-Aldrich Corp. (St. Louis, MO, USA). Genipin (purity > 98%) was purchased from Linchuan Zhixin Biotechnology Co., Ltd. (Fuzhou, China). HCl, NaN_3_, NaOH, and the other unmentioned reagents were of analytical grade.

### 2.2. Extraction of SBP

Pectin was extracted by the conventional hot-acid extraction method according to the report of Yapo et al. [16]. Three SBP samples were extracted with three conditions: (1) pH 1.5, 80 °C, and 1 h (named SBP1); (2) pH 1.5, 80 °C, and 4 h (named SBP2); (3) pH 1.5, 90 °C, and 4 h (named SBP3). The extracted slurry was collected by centrifugation (10,000× *g*, 30 min) and mixed with 3 vol of 95% *v/v* ethanol to precipitated pectin. The precipitated pectin was washed with 95% *v/v* ethanol, dried at 45 °C for 12 h (with water content < 4%), and stored at −20 °C until use.

### 2.3. Genipin-Crosslinking Modification of Pectin

Pectin was modified by genipin-crosslinking according to our previous report [9]. Pectin powder was dispersed into distilled water to set a concentration of 1.5 wt% with magnetic stirring (500 rpm) for 12 h. After adjusting the pH of the pectin solution to 7.5 with HCl or NaOH, genipin powder was added to a concentration of 10 mM and simultaneously initialed the crosslinking reaction. The reaction was performed at room temperature for 25 h and terminated by adding 3 vol of 95% *v/v* ethanol. The precipitated pectin was collected, washed, dried, and stored as stated in Section 2.2.

### 2.4. Composition Analysis

Galacturonic acid (Gal-A) content was determined by the *m*-hydroxydiphenyl colorimetric method [17]. Neutral sugar (NS) content was quantified by high-performance anion-exchange chromatography [18]. Protein content was determined by the Kjeldahl method (N × 6.25) [19]. The ferulic acid (FA) was determined by the colorimetric method [20]. The degree of methylation (DM) and acetylation (DA) were measured by high-performance liquid chromatography [21].

### 2.5. Ultraviolet Spectroscopy

The ultraviolet-visual (UV-Vis) spectrum of pectin solution was measured by a spectrophotometer (TU-1901, PERSEE General Instrument Co., Ltd., Beijing, China). Pectin concentration was set at 1.5 mg/mL and analyzed at room temperature.

### 2.6. Molecular Weight Determination

Size exclusion chromatography (SEC) coupled with multi-angle light scattering (MALLs) detector (DAWN HELEOS, Wyatt Corp., Santa Barbara, CA, USA) and 2414 refractive index (RI) (Waters Corp., Milford, MA, USA) were used to characterize the weight-average molecular weight (Mw) and radius of gyration (Rg) of pectin, according to our previous work [4]. Pectin was dissolved in eluent (100 mM NaNO_3_ with 0.05% NaN_3_ as preservative) at 1 mg/mL and filtered through 0.45 μm filters. The data were analyzed by ASTRA software (version 6.1.1.17, Wyatt Corp., Santa Barbara, CA, USA) with a dn/dc of 0.135 mL/g.

### 2.7. Viscosity Measurement

A Discovery HR-2 rotational rheometer (TA Instrument Co., Ltd., New Castle, DE, USA) was applied for shear viscosity measurement of 1 wt% pectin solution at room temperature with a 40 mm parallel-plate geometry (gap 1 mm). The shear rate was constant at 10 s^−1^ [22].

### 2.8. Emulsions Preparation and Characterization

The emulsion comprised 1 wt% pectin as an emulsifier, 10 wt% MCT as oil phase, and 0.02 wt% NaN_3_ as a preservative. Pectin (1 g) and NaN_3_ (0.02 g) were dissolved into 50 g of citrate buffer (50 mM, pH 3.5) with magnetic stirring (500 rpm) for 12 h. A total of 10 g MCT and a certain amount of citrate buffer were added to the above solution for a total weight of 100 g. The mixture was high-speed sheared (20,000 rpm, 2 min) by a crusher (Slientcrusher M, Heidolph Corp., Schwabach, Germany) for pre-homogenization and subsequently homogenized (NANO, ATS Engineer Inc., Shanghai, China) at 50 MPa for two times to retrieve fine emulsions.

The emulsion droplet size was analyzed by a laser particle analyzer (Mastersizer 3000, Malvern Instruments Ltd., Worcestershire, UK) with a refractive index of 1.45 and 1.33 for MCT and water, respectively. The emulsion stability was evaluated by testing the droplet size changes during storage (55 °C for 5 days) [23].

### 2.9. Photostability Test of Encapsulated β-Carotene in Pectin-Stabilized Emulsions

The *β*-carotene was dissolved in MCT with a concentration of 0.03 wt%. The oil loaded in pectin-stabilized emulsion (pH 6) was increased to 25 wt% and subjected to emulsion preparation as described in Section 2.7. The *β*-carotene-loaded emulsions (20 mL) were placed in a culture dish and exposed to UV lamp radiation (15 W, 302 nm) with 10 cm distance for 10 h at room temperature. After UV radiation, the residual *β*-carotene in the emulsions was recovered by mixing 4.8 mL methanol with 0.4 mL emulsion and separated by centrifugation (10,000× *g*, 5 min) [24]. The content of *β*-carotene was quantified by reversed-phase high-performance liquid chromatography [25].

### 2.10. Statistical Analysis

All experiments were repeated three times, and the data were analyzed by Origin software (OriginPro learning edition, OriginLab Corp., Northampton, MA, USA) with a one-way analysis of variance (ANOVA) and Duncan’s test (*p*-value < 0.05).

## 3. Results and Discussion

### 3.1. Chemical Composition and Macromolecular Characteristics

The chemical composition and macromolecular characteristics of pectin used in this work were shown in Table 1 and Table 2, respectively. The SBPs extracted by different conditions varied greatly in characteristics. As compared with SBP1 extracted under milder conditions (pH 1.5, 80 °C, 1 h), SBPs extracted under harsh conditions for longer time (pH 1.5, 80 °C, 4 h, SBP2) and higher temperature (pH 1.5, 90 °C, 4 h, SBP3) showed substantial degradation in molecular structure as decreasing Mw (from 582.6 to 278.6 kg/mol) and Rg (from 32.1 to 24.7 nm) (Table 2). The harsh condition for extraction significantly increased the pectin yield from 10.42% to 18.93% and Gal-A content from 58.34% to 68.74%, while the content of NS, protein, FA, DM, and DA was significantly decreased (Table 1). It was proposed previously that the resistance of glycosidic linkages inside pectin against acid hydrolysis was increased in order: neutral sugar-neutral sugar < Rha–GalA < Gal–Rha < GalA–GalA [16,26]. The glycosidic linkages in NS side chains were more prone to degradation than the homogalacturonan backbone. Therefore, the protein and FA covalently bound to the NS side chains were simultaneously cleaved from pectin during acid hydrolysis at harsh extraction conditions [8]. Another study has shown that hot acid conditions can decrease DM and DA [27]. Since the intact macromolecular structure and higher protein content usually predicted the good emulsifying properties of pectin [16], compromise should be made to balance the surface activity and yield in industrial production of SBP. Compared with commercial CP and AP, SBP1 (lower degree of degradation) has higher DA, protein, and NS content, agreeing with the result of Liu et al. [3].

Previously, SBP was widely recognized with relatively lower Mw (less than 100,000 kg/mol), which was speculated to impair its gelling ability with a weaker network structure [16,28]. However, the SBP1 in this work and other SBP recently reported by Liu et al. [3] and Niu et al. [29] have significantly larger Mw than the commercial CP and AP (Table 2). These controversial results could be ascribed to the differences in the Mw determination method and the conformation differences between pectin of different sources. The relatively lower Mw of SBP was mainly characterized by intrinsic viscosity [28,30] and SEC coupled with the dextran standards calibration curve [16]. Recently works applied the SEC-MALLs to detect the absolute Mw and Rg of SBP, and it was found that SBP commonly has smaller Rg values but larger values in Mw than CP and AP (similar to the data in Table 2). The unparallel changes in Mw and Rg suggested that the conformation of pectin of different sources might different. Although lower in Mw, the intrinsic viscosity of CP and AP would be detected as larger values than SBP. This is because their larger Rg with more robust intermolecular entanglement (similar apparent viscosity was shown in Table 2), and the calculated viscosity average Mw of SBP would be relatively smaller [28,30]. Generally, the shorter elution time of the tested sample for the SEC experiment indicated its larger Mw (with the same conformation). Since the elution time was dominated by the molecular size (Rg) of samples, the larger Rg of CP and AP would decrease the elution time, thus making the relative calculated Mw of CP and AP larger than SBP, based on the dextran standards calibration curve.

Regardless of the conformation, SEC-MALLs can accurately determine the absolute Mw and Rg based on the light scattering. The differences in conformation were indicated by the values of Mw/Rg in Table 2. The Mw/Rg values of SBP were decreased from 18.1 to 11.3 kg/mol/nm by using harsher extraction conditions, and all three SBPs showed much larger Mw/Rg than CP (8.3 kg/mol/nm) and AP (8.0 kg/mol/nm). It was proposed that SBP with larger Mw but smaller Rg adopted a more compact conformation, which could be explained in two aspects: (1) the higher content of Rha (Table 1) endowed SBP with a more branched structure [31]; (2) the abundant NS side chains with more flexibility than homogalacturonan (HG) made SBP easier to bend and thus efficient packing the polysaccharide chains [32]. Moreover, the cleavage of NS side chains by harsh acid hydrolysis cleaved could substantially decrease the values of Mw/Rg, indicating the SBP2 and SBP3 with lower Rha and NS content adopted a less compact conformation than SBP1.

### 3.2. Genipin-Crosslinking Modification

**Figure 1:**
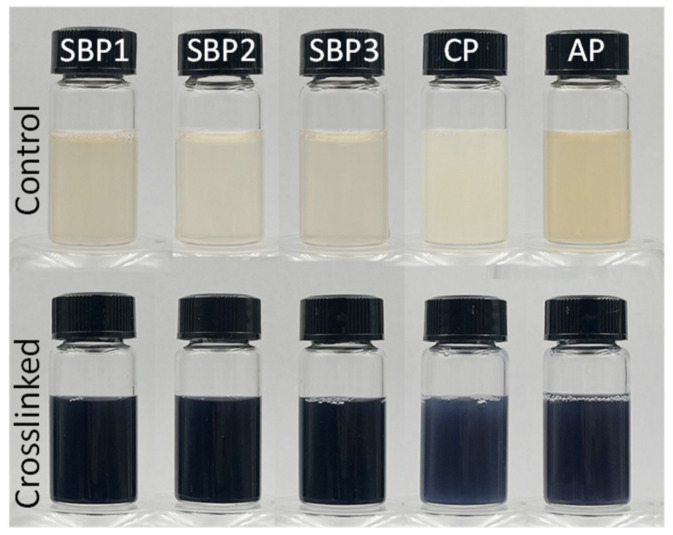
The visual appearance of pectin solution (1 wt%) before (**up**) and after (**down**) genipin crosslinking reaction.

**Figure 2:**
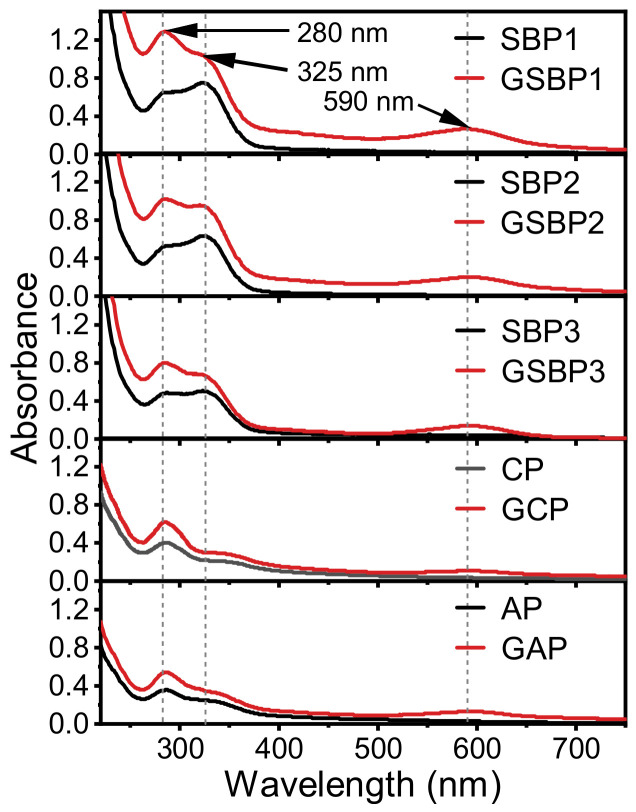
UV-Vis spectra of the pectin before and after genipin crosslinking reaction.

After crosslinking modification by genipin, all 5 tested pectin solutions turned from a slightly yellow to blue-black color (Figure 1). The UV-Vis spectra (Figure 2) depicted that all the control pectin without genipin crosslinking showed absorbance at both 280 and 325 nm, suggesting the presence of protein and phenolic compounds in pectin. The absorbance at 325 nm of three control SBP was ascribed to the FA covalently bound to polysaccharides [33], consisting of the FA content in Table 1. The absorbance at 280 nm was substantially increased after genipin crosslinking, whilst a new absorbance peak appeared at 590 nm. Genipin reacted with two –NH_2_ to produce the heterocyclic amino compounds, contributing to the color and UV-Vis spectra changes [34,35].

The crosslinking reaction was confirmed by determining Mw, Rg, and apparent viscosity (Table 2). Pectin of different sources showed a varying increase in Mw. Regardless of the extraction conditions, the Mw of all the three SBP samples was enlarged more than 2 times after crosslinking, but the increase of Mw was much smaller for CP (1.44 times) and AP (1.31 times). Since the targeted sites of genipin crosslinking were the –NH_2_ in proteinaceous moiety, the Mw was increased only when the –NH_2_ on two independent pectin molecules reacted with the same genipin molecule. Even with lower protein (Table 1), the Mw increase of SBP3 (2.06 times) was larger than that of CP and AP after crosslinking. It could be proposed that not only the protein content influenced the reaction, but also the accessibility of protein played a role in the reaction. As containing more Gal-A and less Rha, the polysaccharide chains in CP and AP were less flexible to bend and likely buried the proteinaceous moiety, hindering their accessibility during crosslinking. Moreover, the apparent viscosity of all pectin samples was significantly increased after genipin-crosslinking modification (Table 2).

### 3.3. Emulsifying Activity

Before emulsion fabrication, the ability to decrease MCT-water interfacial tension was tested and shown in Table 3. As expected, the interfacial tension of three SBP samples was significantly lower than that of CP and AP, implying the better surface activity of SBP and agreeing with the previous report [3,29]. The harsher extraction would impair the surface activity of SBP, in which the interfacial tension of SBP2 and SBP3 was significantly larger than that of SBP1 and could be attributed to the less protein content (Table 1). Although CP (3.15%) and AP (2.78%) contained more protein than SBP3 (2.35%), their interfacial tension was still larger, which was ascribed to the less accessibility of their proteinaceous moiety. After crosslinking, all the crosslinked pectin depicted inferior surface activity as significantly increased interfacial tension. It could be explained by the larger Mw of molecule after crosslinking, which slowed down the diffusion onto the MCT-water interface [1,9].

**Figure 3:**
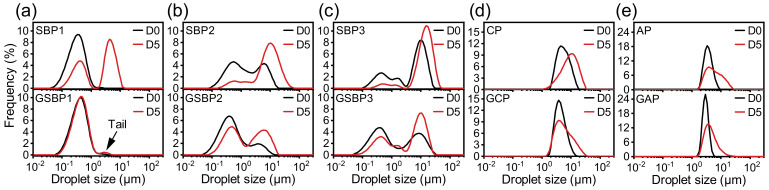
Droplet size distribution of fresh (D0) and stored (D5) emulsions stabilized by SBP1/GSBP1 (**a**), SBP2/GSBP2 (**b**), SBP3/GSBP3 0 (**c**), CP/GCP (**d**), and AP/GAP (**e**).

An emulsion test was performed with 10 wt% MCT as the oil phase and 1 wt% pectin as an emulsifier. The emulsion’s droplet size (*d*_4,3_) was depicted in Table 4. The harsher extraction condition significantly decreased the emulsifying activity of SBP. The *d*_4,3_ of SBP3-stabilized and SBP2-stabilized emulsions (D0, fresh emulsion) were 7.35 and 3.12 μm, respectively, which far exceeded larger than that of SBP-stabilized emulsion (0.36 μm). The good emulsifying activity of SBP1 was also verified by the Gaussian distribution (centered at 0.3–0.4 μm) of its droplet size distribution as compared to the bimodal distribution of that of the emulsions stabilized by SBP2 and SBP3 (Figure 3). These results were consistent with the interfacial tension measurement (Table 3), indicating the degradation of pectin during extraction would greatly impair the emulsifying activity. Therefore, mild conditions should be used for extraction to retrieve SBP with good emulsifying activity [16].

Although the droplet size distribution of CP-stabilized and AP-stabilized emulsions were also the Gaussian distribution as even emulsions (Figure 3c,d), the peaks were centered at ~6 and ~3μm with *d*_4,3_ of 6.09 and 3.79 μm, respectively. The much larger droplet size suggested the inferior emulsifying activity of CP and AP. It was noteworthy that, though the *d*_4,3_ of SBP2 (3.12 μm) and SBP3 (7.35 μm) was similar to those of AP (3.79 μm) and CP (6.09 μm), a large part of the droplets in emulsions stabilized by SBP2 and SBP3 were located in small droplet peaks (centered at 0.3–0.4 μm), which indicated that SBP2 and SBP3 were still able to stabilize a large proportion of small particle size emulsions with better emulsifying activity. The better emulsifying activity of SBP2 and SBP3 may be due to the higher accessibility of their proteins. Although the Mw/Rg value of SBP2 and SBP3 is significantly lower than that of SBP1, it is still larger than CP and AP (Table 2) and contains more NS side chains (Table 1), which are more flexible and easier to bend for contacting the oil-water interface during emulsification.

After genipin-crosslinking modification, only SBP1 showed a decrease in emulsifying activity (*d*_4,3_ increased from 0.36 to 0.45 μm), whereas the *d*_4,3_ of emulsion prepared by the other four crosslinked pectin were all smaller than that fabricated by control pectin (Table 4). The decreasing emulsifying activity of SBP1 after crosslinking was due to the less accessibility of proteinaceous buried by larger bulky polysaccharides chains with larger Mw (Table 2) [9]. For the other four pectins, the enhancement of emulsifying activity was attributed to the thicker adsorbed hydrated layer. Although pectin with smaller Mw could temporarily adsorb onto the oil-water interface during homogenization, the thin adsorbed hydrated layer was prone to breakage and led to the rapid coalescence between droplets, so that the finer droplets are not retained in the final emulsions [2].

### 3.4. Emulsifying Stability

In order to reasonably control the evaluation time of emulsion stability, all prepared emulsions were sealed in a glass vial and stored at 55 °C for 5 days for an accelerated demulsification experiment [36]. Regardless of the differences in sources and extraction conditions, it was found that all the crosslinked pectin showed significant enhancement in emulsifying stability after genipin-crosslinking modification. The changes of *d*_4,3_ of all the crosslinked pectin-stabilized emulsions were far less than that of the emulsions prepared by control pectin (Table 4).

For emulsion stabilized by SBP1, its droplet size distribution was changed from Gaussian distribution to bimodal distribution as a new larger droplet peak appeared centered at ~5 μm (Figure 3a), agreeing with the previous report of undesirable emulsifying stability of SBP [7,8,9]. The emulsions stabilized by GSBP1 depicted excellent stability against storage because the droplet size distribution of stored emulsion was almost overlapped with fresh emulsion with only a tiny trailing peak (Figure 3a). In the case of GSBP2 and GSBP3, the intensity of the small droplet peak (centered at 0.3–0.4 μm) was decreased along with the increase of the large droplet peak (centered at 8–10 μm). The number of large droplets and the shifting degree to larger droplet size direction of stored emulsions stabilized by GSBP2 and GSBP3 were much less than that of SBP2 and SBP3. Similar enhancement of emulsion stability was also observed for emulsions stabilized GCP and GAP. The enhancing emulsifying stability of crosslinked pectin could be explained by the following: (1) the larger Mw (Table 2) endowed a thicker adsorbed hydrated layer at the MCT-water interface, which exerted a stronger steric-hindrance effect to prevent emulsion droplets from coalescence; and (2) larger viscosity (Table 2) of the continuous phase could slow down droplet collision and flocculation [37].

In summary, the above results indicated that the genipin-crosslinking modification could significantly improve the emulsifying stability of pectin. The emulsifying activity was enhanced for the pectin with undesirable emulsification (SBP2 and SBP3 with degradation by acid hydrolysis). These emulsifying properties improvements were applicable to pectin of different sources.

### 3.5. Photostability of Encapsulated β-Carotene in Emulsions

**Figure 4:**
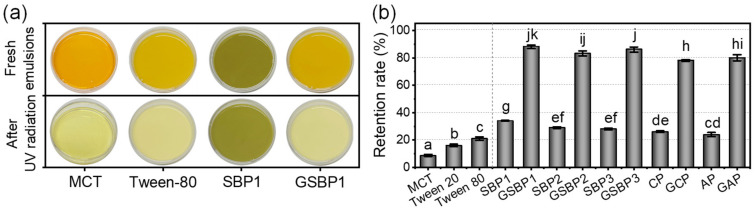
Visual appearance (**a**) and retention rate (**b**) of β-carotene loaded in MCT, Tween-stabilized emulsions, and pectin-stabilized emulsion after 10 h UV radiation. In panel b, the different letters on the column indicate statistically significant differences (*p* < 0.05) among the samples.

As depicted in Figure 1, the crosslinked pectin exhibited a blue-black color and had significant absorbance of UV light, implying its potential for UV-blocking ability. Liang et al. [14] demonstrated a genipin-crosslinking strategy for enhancing montmorillonite-chitosan film, which has an excellent UV-blocking ability to prevent rhodamine B from degradation due to the dark blue pigments. This work loaded *β*-carotene with pectin-stabilized emulsions and exposed it to UV radiation for 10 h at room temperature. The appearance of *β*-carotene-loaded MCT and *β*-carotene-loaded emulsions stabilized by Tween-80 and SBP1 was a yellow color (Figure 4a). The color of GSBP1-stabilized *β*-carotene-loaded showed a dark green color. After UV radiation, a significant fade happened for *β*-carotene-loaded MCT and *β*-carotene-loaded emulsions stabilized by Tween-80 and SBP1. There were no obvious changes in the appearance of *β*-carotene-loaded emulsions stabilized by GSBP1. The fade was attributed to the degradation of *β*-carotene under UV radiation. By quantifying the *β*-carotene retention rate in emulsions (Figure 4b), it was found that the photostability of *β*-carotene-loaded emulsions was enhanced by all the crosslinked pectin regardless of their differences in sources and extraction conditions. The retention rate was only 8.7%, 16.1%, and 21.0% in MCT, Tween-20 emulsion, and Tween-80 emulsion, respectively, suggesting that the loading in emulsions has a weak blocking ability to UV radiation. The retention rate increased to 24.2–34.3% and 78.6–87.3% for *β*-carotene-loaded emulsions stabilized by control pectin and crosslinked pectin. The better UV radiation blocking of pectin-stabilized than Tween-stabilized emulsions was ascribed to the presence of a small number of phenolic compounds (scavenging free radical) and a thicker adsorbed hydrated layer (blocking UV radiation) [12]. The excellent UV blocking ability of crosslinked pectin was contributed by its thicker and denser hydration layer at the MCT-water interface, and its blue substances excellent UV-blocking ability [14].

## 4. Conclusions

The present work aimed to enhance the emulsification and photostability of pectin from different sources and extraction conditions by genipin-crosslinking strategy. Regardless of extraction conditions, SBP has a more compact and flexible conformation than commercial CP and AP, as a more branched structure and more NS side chain content. All the tested pectins were crosslinked by genipin with larger Mw, Rg, and viscosity. The crosslinking modification improved not only all the emulsifying stability of crosslinked pectin but also the emulsifying activity of some pectin with smaller emulsion droplet sizes. The *β*-carotene-loaded emulsions stabilized by crosslinked pectin showed excellent photostability against UV radiation. The retention rate of *β*-carotene in crosslinked SBP1 (20 h crosslinking modification) stabilized emulsion was high at 87.3%. This work demonstrated that the genipin-crosslinking strategy would be feasible to enhance the emulsion stability and be applicable for various pectin.

## Figures and Tables

**Table 1:** Yield and chemical composition of pectin used in this work ^a^.

	SBP1	SBP2	SBP3	CP	AP
Yield (% *w*/*w*)	10.42 ± 0.81a	14.83 ± 0.56b	18.93 ± 1.03c	-	-
Gal-A (% *w*/*w*)	58.34 ± 1.21a	64.25 ± 0.82b	68.74 ± 1.03c	76.23 ± 1.51d	77.65 ± 0.93d
Rha	4.11 ± 0.03e	3.30 ± 0.02d	2.03 ± 0.02c	1.23 ± 0.01b	1.06 ± 0.01a
NS (% *w*/*w*)	24.28 ± 0.40e	19.43 ± 0.31d	15.54 ± 0.24c	13.33 ± 0.13b	12.15 ± 0.10a
Protein (% *w*/*w*)	6.72 ± 0.21d	3.76 ± 0.12c	2.35 ± 0.06a	3.15 ± 0.08b	2.78 ± 0.04b
FA (% *w*/*w*)	0.96 ± 0.06c	0.65 ± 0.03b	0.48 ± 0.05a	n. a. ^b^	n. a.
DM (%)	53.41 ± 0.53c	42.63 ± 1.08b	38.17 ± 0.76a	52.42 ± 1.15c	67.62 ± 1.32d
DA (%)	29.32 ± 0.54e	22.55 ± 0.26d	17.24 ± 0.27c	1.49 ± 0.06a	3.6 ± 0.03b

GalA, galacturonic acid; Rha, rhamnose; NS, neutral sugar (the sum of rhamnose, arabinose, galactose, glucose, and xylose); FA, ferulic acid; DM, degree of methylation; and DA, degree of acetylation. ^a^ Data are means ± standard deviations (*n* = 3). Values in the same row followed by different letters are significantly different (*p* < 0.05). ^b^ n.a., not applicable.

**Table 2:** Molecular weight (Mw) and radius of gyration (Rg) and apparent viscosity (η) of the pectin before and after genipin crosslinking ^a^.

	Mw (kg/mol)		Rg (nm)		Mw/Rg	η (mPa·s)	
	Control	Crosslinked	Control	Crosslinked	(kg/mol/nm)	Control	Crosslinked
SBP1	582.6 ± 23.1d	1441.6 ± 32.7e *	32.1 ± 1.2c	37.2 ± 0.9c *	18.1 ± 0.2e	14.12 ± 0.18c	23.2 ± 0.18c *
SBP2	392.1 ± 17.2c	834.3 ± 26.8d *	28.5 ± 0.8b	31.3 ± 0.3b *	13.8 ± 0.2d	11.26 ± 0.22b	17.3 ± 0.13b *
SBP3	278.6 ± 13.4a	573.5 ± 19.3c *	24.7 ± 1.0a	27.4 ± 0.5a *	11.3 ± 0.2c	9.53 ± 0.16a	13.5 ± 0.09a *
CP	324.5 ± 12.3b	468.3 ± 22.4a *	39.3 ± 1.6d	43.2 ± 1.2d *	8.3 ± 0.1b	26.38 ± 0.32d	37.6 ± 0.24d *
AP	376.7 ± 16.8c	493.3 ± 15.7b *	46.8 ± 2.1e	53.8 ± 1.8e *	8.0 ± 0.1a	39.24 ± 0.42e	51.8 ± 0.38e *

^a^ Data are means ± standard deviations (*n* = 3). Values in the same column followed by different letters are significantly different (*p* < 0.05). The values of crosslinked samples followed by a star (*) for Mw, Rg, and η indicated a significant difference (*p* < 0.05) as compared to its control samples.

**Table 3:** Interfacial tension (mN/m) of pectin at MCT-water interface ^a^.

	SBP1	SBP2	SBP3	CP	AP
Control	12.30 ± 0.38aA	14.31 ± 0.16bA	17.63 ± 0.13cA	22.18 ± 0.34eA	19.56 ± 0.26dA
Crosslinked	14.14 ± 0.14aB	15.15 ± 0.19bB	18.01 ± 0.09cB	23.23 ± 0.15eB	20.75 ± 0.21dB

^a^ Data are means ± standard deviations (*n* = 3). Values in the same row followed by different small letters and in the same column followed by different capital letters are significantly different (*p* < 0.05).

**Table 4:** Yield and chemical composition of pectin used in this work ^a^.

	D0 (μm)		D5 (μm)		Changes (μm)	
	Control	Crosslinked	Control	Crosslinked	Control	Crosslinked
SBP1	0.36 ± 0.01a	3.15 ± 0.11d	0.45 ± 0.01b	0.51 ± 0.01c	2.79	0.06
SBP2	3.12 ± 0.04b	10.32 ± 0.31d	1.16 ± 0.03a	4.24 ± 0.12c	7.20	3.08
SBP3	7.35 ± 0.14c	17.27 ± 0.48d	3.61 ± 0.09a	4.87 ± 0.13b	9.92	1.26
CP	6.09 ± 0.21b	10.64 ± 0.35d	5.12 ± 0.05a	7.06 ± 0.15c	4.56	1.94
AP	3.79 ± 0.03b	6.85 ± 0.21d	3.01 ± 0.01a	4.52 ± 0.08c	3.06	1.51

GalA, galacturonic acid; Rha, rhamnose; NS, neutral sugar (the sum of rhamnose, arabinose, galactose, glucose, and xylose); FA, ferulic acid; DM, degree of methylation; DA, degree of acetylation. ^a^ Data are means ± standard deviations (*n* = 3). Values in the same row followed by different letters are significantly different (*p* < 0.05).

## Data Availability

Data is contained within the article.
